# Effectiveness and cost-effectiveness of an educational intervention for practice teams to deliver problem focused therapy for insomnia: rationale and design of a pilot cluster randomised trial

**DOI:** 10.1186/1471-2296-10-9

**Published:** 2009-01-26

**Authors:** A Niroshan Siriwardena, Tanefa Apekey, Michelle Tilling, Andrew Harrison, Jane V Dyas, Hugh C Middleton, Roderick Ørner, Tracey Sach, Michael Dewey, Zubair M Qureshi

**Affiliations:** 1 Faculty of Health, Life & Social Sciences, University of Lincoln Brayford Pool, Lincoln, LN6 7TS, UK; 2 NHS Lincolnshire, Cross O'Cliff Court, Bracebridge Heath, Lincolnshire, LN4 2HN, UK; 3 Linking Voices, Church Lane, Sleaford, Lincolnshire, NG34 7DF, UK; 4 National Institute for Health Research, Research Design Service East Midlands, Tower Building, University Park, Nottingham, NG7 2RD, UK; 5 School of Sociology and Social Policy, University of Nottingham, University Park, Nottingham, NG7 2RD, UK; 6 Somerby Clinic, 8 Lindum Terrace, Lincoln, LN2 5RS, UK; 7 School of Chemical Sciences and Pharmacy, Health Economics Group, University of East Anglia, Norwich, NR4 7TJ, UK; 8 Health Service and Population Research, Institute of Psychiatry, Box P060, De Crespigny Park, London, SE5 8AF, UK

## Abstract

**Background:**

Sleep problems are common, affecting over a third of adults in the United Kingdom and leading to reduced productivity and impaired health-related quality of life. Many of those whose lives are affected seek medical help from primary care. Drug treatment is ineffective long term. Psychological methods for managing sleep problems, including cognitive behavioural therapy for insomnia (CBTi) have been shown to be effective and cost effective but have not been widely implemented or evaluated in a general practice setting where they are most likely to be needed and most appropriately delivered. This paper outlines the protocol for a pilot study designed to evaluate the effectiveness and cost-effectiveness of an educational intervention for general practitioners, primary care nurses and other members of the primary care team to deliver problem focused therapy to adult patients presenting with sleep problems due to lifestyle causes, pain or mild to moderate depression or anxiety.

**Methods and design:**

This will be a pilot cluster randomised controlled trial of a complex intervention. General practices will be randomised to an educational intervention for problem focused therapy which includes a consultation approach comprising careful assessment (using assessment of secondary causes, sleep diaries and severity) and use of modified CBTi for insomnia in the consultation compared with usual care (general advice on sleep hygiene and pharmacotherapy with hypnotic drugs). Clinicians randomised to the intervention will receive an educational intervention (2 × 2 hours) to implement a complex intervention of problem focused therapy. Clinicians randomised to the control group will receive reinforcement of usual care with sleep hygiene advice. Outcomes will be assessed via self-completion questionnaires and telephone interviews of patients and staff as well as clinical records for interventions and prescribing.

**Discussion:**

Previous studies in adults have shown that psychological treatments for insomnia administered by specialist nurses to groups of patients can be effective within a primary care setting. This will be a pilot study to determine whether an educational intervention aimed at primary care teams to deliver problem focused therapy for insomnia can improve sleep management and outcomes for individual adult patients presenting to general practice. The study will also test procedures and collect information in preparation for a larger definitive cluster-randomised trial. The study is funded by The Health Foundation.

**Trial Registration:**

ClinicalTrials.gov ID ISRCTN55001433 –

## Background

Insomnia and sleep problems are common, affecting over a third of adults in the United Kingdom.[1,2] Many of those whose lives are affected seek medical help from primary care.[3] A review of cost effectiveness studies of diagnosis and treatment of insomnia highlighted that insomnia has a high economic cost on society, particularly in terms of productivity loss and impaired health-related quality of life.[4]

General practitioners and nurse prescribers have been shown to have a limited repertoire of responses (sleep advice sheet, prescription) when managing new patients or reviewing those with insomnia. Clinical responses are shaped by unduly positive attitudes towards newer hypnotics[5] and limited by lack of knowledge of alternative non-pharmacological approaches. This leads to management strategies that often involve the prescription of hypnotic drugs, despite the finding that GPs hold less than favourable views of these drugs overall.[6] Evidence also suggests that hypnotic prescribing fosters patient dependency and passive 'help seeking behaviour' involving further requests for prescriptions. The clinical benefits of hypnotics are small and short-lived. They also carry significant risks of adverse events, for example increases in risk of falls, fractures,[7] depression[8], suicide[9] and excess mortality.[10] These dangers are particularly pronounced in the elderly who are most vulnerable to potential harm.[11] A compelling case exists for both improving quality of care for these patients and doing so by encouraging changes in prescribing, although there is less evidence about how to achieve these outcomes.

We could find no published evidence on the costs of poor sleep in the UK. However in the US the costs (direct, indirect and related) of insomnia have been estimated at US$30 to 35 billion per annum (1994 US$) [12] and in Australia AUS$7494 million (2004 AUS$) of which 53% was accounted for by work-related injuries (net of health costs), private motor vehicle accidents (net of health costs) and productivity losses associated with sleep disorders.[13] The review by Martin et al.[4] made three research recommendations encompassing the need for more accurate costs for patients with different types of insomnia related diagnosis, an estimate of the health utilities associated with particular insomnia diagnoses and their treatments and finally to undertake cost utility analyses of treatments for insomnia to encourage resource allocation to this area.

We have shown in modelling studies involving patients, practitioners and practices that despite patients trying various self-help remedies and sometimes expecting a prescription when presenting with sleep problems, they also welcome a consultation approach which includes careful assessment and advice about non-pharmacological methods rather than a prescription. [14-16] Practitioners are generally positive towards finding new approaches that enhance their skills in non-pharmacological management methods. Brief psychological/behavioural methods[17] have also been shown to be effective in one previous limited evaluation of effectiveness.[18]

The objective of this paper is to describe the design of a pilot cluster randomised controlled study, the aim of which is to test procedures and collect information in preparation for a larger definitive cluster-randomised trial. The definitive study will determine the effectiveness and cost-effectiveness of an educational intervention for general practitioners, primary care nurses and other members of the primary care team to deliver problem focused therapy to adults patients presenting with sleep disturbance due to primary insomnia or specific comorbid insomnia due to pain or mild to moderate depression and anxiety compared to usual care (general advice on sleep hygiene and pharmacotherapy).

The team includes a general practitioner, psychologist, psychiatrist, qualitative researcher, statistician and health economist and a service user representative who have worked together undertaking studies of sleep management in primary care and have contributed to the rationale and design of this study.

## Methods

### Aims

Our main aim for the pilot study is to test procedures and collect information in preparation for a larger definitive cluster-randomised trial. The definitive trial will investigate the effectiveness and cost-effectiveness of a brief practice-based educational intervention (2 × 2 hours) for practice teams (GPs, primary care nurses and practice managers) to provide problem focused therapy for insomnia in adults involving the use of sleep assessment tools (assessed with standardised instruments and sleep diaries) and modified CBTi on sleep outcomes compared to usual care (general advice on sleep hygiene and pharmacotherapy). The study will include new patients presenting with primary and specific comorbid insomnia as well as patients with chronic insomnia on treatment.

The research question for the pilot trial is "How well do the procedures and processes designed for conduct of the trial lead to effective implementation of trial including recruitment, randomisation, intervention, and outcome measurements."

The research question for the main trial is as follows "How effective and cost-effective is an educational intervention to primary care teams (general practitioners, primary care nurses and practice managers) to deliver problem focused therapy to adult patients presenting with sleep problems due to lifestyle causes, pain or mild to moderate depression on sleep and quality of life compared with treatment as usual including sleep hygiene and pharmacotherapy?"

The primary hypothesis for the main trial to be addressed is:

"Education for primary care teams in problem focused therapy for patients presenting to primary care with insomnia leads to better sleep outcomes for patients compared to treatment as usual with sleep hygiene up to 3 months from the beginning of treatment."

Secondary hypotheses for the main trial include: "Education for primary care teams in problem focused therapy for patients presenting to primary care with insomnia improves daytime sleepiness, anxiety and depression, health-related quality of life and reduces hypnotic use, adverse effects and costs compared to treatment as usual including sleep hygiene."

### Study design

This study will be a pilot in preparation for a larger cluster-randomised trial. The purpose of undertaking this pilot will include testing the integrity of study protocol, recruitment and consent issues, outcome selection, data collection procedures, the randomisation procedure, and the acceptability of the intervention and determining a more accurate sample size.

**Figure 1:**
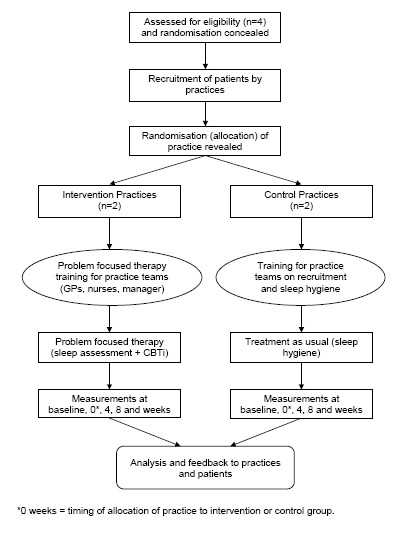
**Consort diagram**.

A flow diagram summarizing recruitment and follow-up of physicians and patients is shown in Figure 1. We will undertake a pilot cluster randomised controlled trial (RCT) in which general practices are the unit of randomisation and where data will be collected from patients. Recruited general medical practices will be randomised to one of two arms: *intervention *consisting of education of primary care teams to use Problem Focused Therapy for insomnia comprising sleep assessment tools (sleep diaries and Insomnia Severity Index) and modified CBTi (mCBTi); or a *control *arm using treatment as usual (TAU). We will follow the CONSORT statement extension on conduct and reporting of cluster randomised controlled trials.[19]

### Participants

#### The Practices

We will recruit four practices from Lincolnshire Teaching Primary Care Trust using EMIS or SystemOne computer systems. Each practice will need to recruit at least 20 patients, based on the sample size calculation for a definitive RCT who meet the inclusion criteria in order to achieve the estimated sample size of 80 patients. Practices will be randomised to intervention and control groups once the practices have been recruited but allocation will be concealed until after patients have been recruited in order to reduce bias in selection and recruitment of patients to intervention and control practice.[20] Invitation letters will be sent out to all practices in Northwest Lincolnshire (40 practices) inviting participation. Patients will be recruited from practices that agree to participate.

#### Study population

Intervention and controls patients will include adults aged 18 years and over with primary or specific secondary (comorbid) insomnia having a Pittsburgh Sleep Quality Index (PSQI) of 5 or above and screened by a trial nurse for the following inclusion and exclusion criteria[21].

#### Inclusion criteria

1. At least 18 years old.

2. Difficulty initiating and/or maintaining sleep for 1 month or more verified by PSQI score of ≥5.

3. New presentations with insomnia and existing patients on long term hypnotics, over the counter or complementary therapies.

4. 1–3 above and sleep disrupted by painful conditions.

5. 1–3 above and depression measured by the Beck Depression Inventory (BDI score 11–30).

#### Exclusion criteria

1. Current or previous illicit substance or alcohol abuse.

2. Pregnant or planning pregnancy in the next 12 months

3. Psychotic illness and severe depression defined by a BDI score ≥ 31.

4. Documented or active symptoms of sleep disruptive comorbid conditions e.g. restless legs syndrome and any type of parasomnia.

5. Obstructive sleep apnoea.

6. Terminal Illness.

7. Inability to consent.

8. Current participation in another research study.

#### Participant recruitment

**Figure 2:**
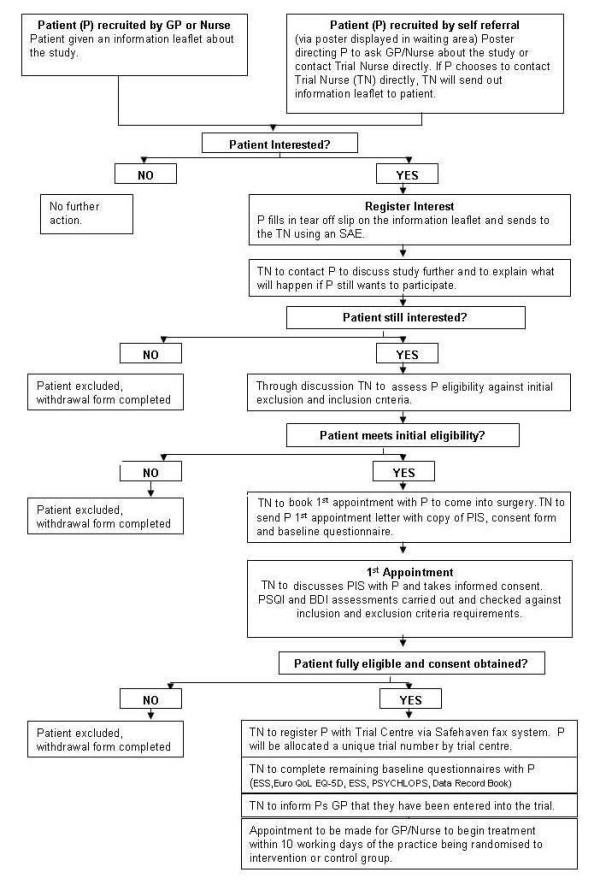
**Participant recruitment flowchart**.

Eligible patients will be recruited by the GP, practice nurse or self referred (via a poster in the GP waiting room). After reading an information leaflet potential participants will register their interest to take part in the trial using a tear-off slip (see Figure 2).

At least 48 hours after and within 10 working days of registering their interest, patients will be telephoned by the trial nurse (this is a practice based nurse trained to recruit and consent patients) to explain the study and check whether they still wish to participate. If the patient is interested the trial nurse will assess the patient against the above listed inclusion and exclusion criteria. Information will be recorded on a standard proforma including, for patients either not eligible or not willing to be recruited, the reason for ineligibility or for declining. Patients meeting the criteria will be sent an invitation by post to an initial assessment visit together with a copy of the Patient Information Sheet (PIS), consent form and baseline demographic questionnaire.

When the patient attends for the initial assessment a (practice based) trial nurse will discuss the PIS with the patient and gain informed consent. The PSQI and BDI will be carried out and checked against the inclusion and exclusion criteria requirements.

If the patient is fully eligible and informed consent is obtained the participant will be entered into the trial. The trial nurse will register the patient with the Trial Centre via a Safehaven fax system. The patient will be allocated a unique trial number by the trial centre. The trial nurse will inform the patient's GP that they have been entered into the trial. The rest of the baseline assessments will be performed at this stage (EuroQol EQ-5D, ESS, ISI, Sleep Diary, and PSYCHLOPS). An appointment will be made for the GP or practice nurse to begin assessment/treatment within 10 working days of the practice being allocated to either the intervention or control group or in the intervention practices, after training of the practice teams on problem focused therapy for insomnia.

Recruitment is planned to take place over 3 months using the methods described above including posters in GP waiting rooms and reminders to GPs and nurses supported by an information leaflet. During the recruitment phase patients will continue to receive treatment as usual.

### Randomisation and allocation concealment

Participating practices will be numbered and randomised to two groups using a computer-generated list of practice identifiers and these will be passed on to the statistician (who will be an investigator remote from the study site) blind to the identity of the practices and using random numbers to ensure allocation concealment. The list will be returned to trial staff, who will break the code and identify the practice allocation when patient recruitment is complete. This information will not be returned to the statistician (MD) so that he will remain masked to treatment allocation while undertaking the analysis. Because of the nature of the study it will not be possible to mask the practices or patients to the intervention but we shall take all possible steps to conduct the analysis masked to treatment.

### Intervention general practices

The intervention will consist of two two-hour educational sessions, using a previously published model of academic detailing to primary care teams.[22] GPs, practice nurses and practice manager will be invited to participate. The importance of all members of the team, including practice managers being involved is due to the key need, demonstrated in the modelling studies, for management support in developing the pathway of care within practices. The first session will include the background to the study, trial structure and procedures, assessment of sleep problems including the use of sleep assessment tools and measurements and information on sleep hygiene. The second session will be on the use of problem focused therapy in the consultation which includes careful assessment (using assessment of secondary causes, sleep diaries and severity) and modified cognitive behavioural therapy for insomnia. This session will also involve practice teams designing their own pathway for care. The timing and number of educational sessions is based on previous modelling studies with a group of 8 practices.

### Control general practices

The control practices will also receive the first session of training which will include the background to the study, trial structure and procedures, assessment of sleep problems including the use of sleep assessment tools and measurements and information on sleep hygiene. They will continue their usual practice (treatment as usual) supported by a standard sleep hygiene leaflet. In the UK, treatment as usual consists of advice on sleep hygiene (with or without a patient information leaflet) and hypnotic pharmacotherapy. We will provide advice on sleep hygiene and a sleep hygiene leaflet for control practices. Sleep hygiene consists of a good bedtime routine, avoiding alcohol, caffeine and nicotine and ensuring a comfortable sleeping environment.

### Audit of interventions and activity

Primary care teams in the intervention practices will be required to send all clinicians and the practice manager to the educational sessions. Further educational sessions will be provided to clinical staff newly recruited during the study period as the need arises. GPs and nurses in intervention and control practices will record patients who present with insomnia and their use of sleep assessment and problem focused therapy techniques using practice computer systems. The content and usefulness of training will be assessed using questionnaires to those in receipt of training. Intervention and control practices will be audited on implementation of the educational intervention by measuring use of assessment and CBTi techniques used by undertaking a weekly computer Read codes audit of all patients recorded with insomnia as well as asking patients through telephone interviews. A sample of up to 4 patients and practitioners from each practice will be invited for a short telephone interview (20 minutes) to understand in more depth the interventions that have been delivered and received as well as perceptions towards these. The interview will determine fidelity to the intervention in study practices and contamination in the control practices and therefore will not be blinded.

### Outcomes

The ***primary outcome measure ***will be global sleep quality as measured by PSQI at baseline and at 0, 4, 8 and 13 weeks.

***Secondary outcomes ***will be measured at baseline and at 0,4,8 and 13 weeks and will include the effect of the intervention on sleep outcome measures assessed with PSQI and sleep diaries [23]: self reported Sleep Onset Latency (SOL, how long it takes to fall asleep), Wake time After Sleep Onset (WASO, total hours awake at night after one has fallen asleep), total Time In bed (TIB) and Sleep Efficiency (SE). Sleep Efficiency, expressed as a percentage, is calculated as follows: SE = (100-[(SOL + WASO/TIB) × 100]). Research has shown that these subjective measures of sleep reflect subjective treatment-related improvements.

We will also measure daytime sleepiness (Epworth sleepiness scale),[24] anxiety and depression using the Beck Depression Inventory,[25] health-related quality of life using EuroQol EQ-5D,[26] psychological outcome profile (PSYCHLOPS)[27] and frequency of use and mean dose of hypnotic medication. Patients will also keep a Data Record Book (DRB) to record any adverse effects that they might experience during the treatment period. Cost-effectiveness will be assessed using the methods below.

### Follow-up

Primary and secondary outcomes will be measured using the instruments described at baseline and 0, 4, 8 and 13 weeks. Follow-up assessments will be performed using a telephone call at 2 weeks after the first treatment and self-completed postal questionnaires at all other time points. Non responders will be telephoned 1 week after mailing the follow-up questionnaire on up to 2 occasions and posted a replacement questionnaire with a reminder letter if there is still no response at 2 weeks.

### Criteria and procedure for withdrawal of patients from the trial

A patient may be withdrawn or themselves withdraw from the trial at any time, at his/her own discretion or at that of the GP or researcher if there is any suspicion of adverse effects or an exclusion condition (including loss of capacity to consent) supervening. The reasons for interrupting the patient's participation in the trial will be recorded on the summary page of the Data Record Book (DRB) and the patient ' proforma. His/her GP will be notified of the need for clinical follow up.

### Justification of sample size

Assuming an intracluster correlation coefficient (ICC) of 0.01 we need at least 15 practices per arm and a cluster size of 20 (i.e. 20 patients in each practice) giving 300 patients per arm to detect a difference of 1 point in the PSQI with 90% power for a definitive RCT. This would require a major investment of resources and therefore we will first conduct an exploratory trial to understand the feasibility of a full trial. For our exploratory trial we will take a pragmatic decision to include 4 practices, 2 intervention and 2 control practices.

### Methods of data collection

Patient level data will be collected from patients using self-report questionnaires, and researchers by telephone. Outcomes will be measured at baseline and at week 0, at 4 weeks, and at 8 and 13 weeks from the initial treatment whether treatment has been completed or not (i.e. on an intention to treat basis).

### Data analysis

Data collection and statistical analysis will be undertaken on both clustered data and on individual patient data masked to treatment allocation and on an intention to treat basis. Data will be analysed at baseline, at 0, 4, 8 and 13 weeks. Baseline clinical and demographic data will be analysed for important differences between trial groups. Comparisons between treatment arms before and after intervention will be made using proportions and odds ratios. We shall analyse the primary outcome using linear regression with baseline as a covariate and also controlling for other relevant baseline covariates. We shall analyse our primary outcome, the PSQI, at the level of the patient using a multivariable model which adjusts for baseline PSQI and for the clustering due to practices. We shall carry out similar analyses for our secondary outcomes. In addition we shall extend our model to incorporate all the time points. This will be a mixed effects model with random effects for time and practice. This model has the advantage that sporadic missing data can be coped with if it is missing at random (MAR). If there is substantial dropout we shall examine it using a pattern mixture model which does not have the restriction to MAR. We shall conduct analogous analyses for the other secondary outcomes. Statistical significance will be set at 5% (two tailed) and analysis performed using Stata or other statistical software. No interim analysis of the primary or secondary outcomes will be undertaken during the trial period.

Template analysis will be used to undertake a qualitative analysis of a short telephone interview (20 minutes) to investigate perceptions of practitioners and patients on interventions delivered and received in each practice with 4 patients and 4 practitioners from each intervention and control practice.

### Health economic analysis

Since a cost analysis will be undertaken for the main trial and the instruments and methods will be piloted in this study, the rationale and methods are therefore described below. Given the high cost of insomnia and the need for economic research in this area, the economic evaluation for the main study will estimate the cost-utility of a brief educational intervention for primary care teams (GPs, nurses and practice managers) to improve sleep management using problem focused therapy (including CBTi) compared to usual care provided by GPs and nurses from a health and social care perspective in the base case. The wider costs will be captured separately. Established and accepted economic methodologies will be employed throughout.[28,29]

Resource items likely to change as a result of the intervention (as well as the cost of developing and providing the educational intervention) will be identified, measured and valued drawing upon instruments used in other studies and available from published resources.[30] The base case will capture only those costs incurred by the NHS and social care (including, for instance, patient visits to health and social care professionals, outpatient visits and admissions to secondary care, and medication) using patient level data extracted from self completion questionnaires administered at baseline, 0, 8 and 13 weeks. This will include all health and social care resource use (due to the difficulties of disentangling) which are consumed as a result of sleep problems per se rather than because of other causes. Where feasible the wider costs associated with sleep problems will be monitored through patient questionnaires, in particular we will attempt to estimate patient costs, particularly associated with health care usage (for instance, over the counter medication and travel costs) and the productivity costs (both absenteeism and presenteeism) [31,32] of patients, whether due directly to sleep problems or accidents, to see if the intervention has any impact on the patient's wider well-being. Unit costs will be derived from national published data (for instance, NHS reference costs,.[33] Annual Survey of Hours and Earnings (ASHE)) for the most recent price year available or local unit costs will be estimated where appropriate and feasible.

This study will undertake cost-effectiveness and cost utility analyses. Both types of analyses enable technical efficiency questions to be addressed, that is to ask, is an educational intervention for health professionals a cost effective way in which to improve the management of patients with sleep problems? In addition, cost-utility analysis will also enable allocative efficiency questions to be addressed within the health sector, for instance, is the brief educational intervention about CBTi considered in this study cost-effective compared to a diverse range of other health interventions or services? This study will estimate the cost-effectiveness in terms of cost per 1 point change on the Pittsburgh Sleep Quality Index (PSQI) and the cost utility using the EuroQol EQ-5D [26] to estimate the cost per QALY over the trial period. Both outcome instruments will be administered at baseline and 13 weeks to observe if change occurs as a result of the intervention and whether it is sustained.

The timeframe for the economic analysis will be that of the trial period. The change in costs will be divided by the change in effectiveness in order to estimate the cost effectiveness of the brief educational intervention compared to no brief educational intervention for health professionals. If non-dominance occurs (that is if costs are greater and the intervention is more effective or if the intervention is cheaper and less effective) an incremental cost-effectiveness ratio (the ratio of change in cost divided by change in benefit) will be produced. The confidence region around the incremental cost effectiveness ratio will be estimated using appropriate statistical techniques. Since this economic evaluation is being undertaken alongside a cluster randomised trial the analysis needs to reflect the increased uncertainty of randomising clusters rather than individuals, a number of approaches have recently been proposed each found to generate similar findings.[34] The stochastic analysis will enable a cost effectiveness acceptability curve to be produced [35] illustrating the decision uncertainty, that is the probability that the brief educational intervention for health professionals on improving sleep management is cost-effective compared to no educational intervention/usual care for a range of willingness to pay per QALY values.

### Ethical approval

The study has been approved by the National Research Ethics Service (REC reference number: 08/H0401/89) and NHS Lincolnshire (Trent) Research Governance. All data will be stored confidentially in encrypted files. Practices will give informed written consent and individual patient consent will also be needed. Data will be kept securely for 13 years.

## Discussion and conclusion

The objective of this study is to test the procedures for a definite trial investigating the effectiveness and cost-effectiveness of an educational intervention for general practitioners, primary care nurses and other members of the primary care team to deliver problem focused therapy to adult patients presenting with sleep problems and anxiety compared with usual care (general advice on sleep hygiene and/or sleeping tablets). Problem focused therapy includes a consultation approach comprising careful assessment (using assessment of secondary causes, sleep diaries and severity) and the use of modified CBTi for insomnia.

This trial is designed to test procedures and collect data in preparation for a larger definitive cluster-randomised trial which will investigate the effectiveness and cost-effectiveness of a brief practice-based educational intervention (2 × 2 hours) for practice teams (GPs, primary care nurses and practice managers) to provide problem focused therapy for adults presenting with sleep problems in primary care due to lifestyle causes, pain or mild to moderate depression. It will include new patients presenting with primary and specific comorbid insomnia as well as patients with chronic insomnia on treatment.

The intervention was developed using the theoretical and modelling phases of the Medical Research Council Framework for complex interventions.[36,37] We are conducting this trial in a primary care population using a team based educational intervention which has been used previously.[22]

This trial will form the basis of a definitive cluster trial of a carefully modelled psychological intervention for patients presenting with sleep problems to be managed in a primary care setting. If problem focused therapy is effective and safe when delivered by primary care clinicians this will have a potential impact for sufferers who are often inappropriately prescribed drugs long term with evidence of more harm than benefit in the elderly. The study would have the potential of providing evidence to improve management of insomnia for a large number of sufferers and thereby to improve the well-being, mental and physical health of many people with sleep problems. If a definitive trial shows that such an intervention is effective and cost-effective this would provide valuable information to general practitioners, mental health trusts and commissioners for provision of such services. If the treatment is shown to be ineffective or costly then this will also provide valuable information about whether such treatment should be provided.

## Competing interests

The authors declare that they have no competing interests.

## Authors' contributions

ANS led the grant-writing group. All authors were involved in the development and application of the protocol. ANS is the guarantor of this paper.

## Pre-publication history

The pre-publication history for this paper can be accessed here:


